# Not all screens are equal: associations between screen-based sedentary behavior and physical activity in Chinese children and adolescents

**DOI:** 10.3389/fpubh.2025.1681183

**Published:** 2025-11-19

**Authors:** Yuan Wang, Binping Gong, Junkai Zhang, Wenle Chen, Mingming Guo

**Affiliations:** 1 Medical Research Center, First Affiliated Hospital of Zhengzhou University, Zhengzhou, Henan, China; 2 Department of Physical Education, College of Education for the Future, Beijing Normal University, Zhuhai, Guangdong, China

**Keywords:** screen-based sedentary behavior, physical activity, children and adolescents, subgroup differences, China Family Panel Studies (CFPS)

## Abstract

**Background:**

Prior research has produced mixed results on the association between screen-based sedentary behavior (SSB) and physical activity (PA) in children and adolescents. Limited attention has been paid to how different types of SSB relate to PA across subgroups.

**Methods:**

This study analyzed data from 2,328 Chinese children and adolescents (aged 10–18) in the 2020 and 2022 waves of the China Family Panel Studies. Sufficient PA was defined as at least 60 min per session, five times a week. SSB types included online gaming, online shopping, short video watching, online learning, and WeChat use, categorized into never, occasional, and daily use. Logistic regression was used to explore associations between SSB and insufficient PA, with subgroup analyses by gender, educational level, and urban-rural residence.

**Results:**

Daily short video watching was associated with higher odds of insufficient PA among females (OR = 1.68), while occasional watching also increased the risk among elementary school students (OR = 1.61). Rural children and adolescents who occasionally engaged in online learning were more likely to report insufficient PA compared with those who never participated (OR = 1.32). In contrast, WeChat use was associated with lower odds of insufficient PA, particularly among males (OR = 0.19), rural children and adolescents (OR = 0.64), and junior high school students (OR = 0.59). No significant associations were found between online gaming or online shopping and insufficient PA.

**Conclusions:**

Different types of SSB show divergent associations with PA across subgroups. Short video watching and online learning may hinder PA, whereas moderate WeChat use appears to support it. Tailored, subgroup-specific intervention ns are needed to mitigate the risks of SSB and promote PA and health among children and adolescents.

## 1 Introduction

Physical activity (PA)—any bodily movement produced by skeletal muscles that expends energy (1)—is critical for children's and adolescents' physical and mental development, benefiting fitness, cognition, emotion regulation, social skills and academic outcomes (2, 34, 35). Despite the World Health Organization's recommendation that children and adolescents engage in at least 60 min of moderate-to-vigorous physical activity (MVPA) daily (3), global surveillance shows that only a minority of this population meets this guideline. In particular, PA levels among Chinese children and adolescents are especially low (4, 36). Identifying modifiable correlates of PA is therefore an urgent public health priority.

Screen-based sedentary behavior (SSB)—defined as time spent using devices such as smartphones, tablets, computers, and televisions—has been widely implicated in reduced PA among children and adolescents. However, existing evidence remains inconsistent: while some studies suggest that certain forms of SSB (e.g., social media use) displace real-world activity and reduce PA, other findings indicate that screen activities involving social interaction may promote or co-occur with higher PA (5, 37). One plausible explanation for these inconsistencies is that SSB is a heterogeneous construct. Different screen activities differ in purpose (educational vs. recreational), interactivity (passive viewing vs. interactive play), and social function (private consumption vs. social communication), and these qualitative distinctions may produce divergent associations with PA. Yet, this hypothesis has not been systematically tested to date. Furthermore, although a considerable body of research has examined the overall relationship between SSB and PA, no studies have explicitly investigated whether these associations differ across key subgroups, such as gender, educational level, or urban–rural residence. These gaps hinder the development of precise, subgroup-tailored interventions to mitigate the adverse effects of SSB (6).

To address these research gaps, the present study drew on data from two waves of a nationally representative cross-sectional survey to examine the associations between five distinct types of SSB—online gaming, online shopping, short video watching, online learning, and WeChat use—and PA among Chinese children and adolescents. Furthermore, the study explored whether these associations varied by gender, educational level, and urban–rural residence. In addition, two hypotheses were proposed: (1) the associations between SSB and PA differ by the type of SSB, and (2) these associations vary across demographic subgroups of children and adolescents. The findings aim to provide empirical evidence to guide policies and interventions on screen use and PA promotion, ultimately helping balance the benefits of digital technology with the need to maintain a healthy and active lifestyle.

## 2 Materials and methods

### 2.1 Data source and sample composition

This study used data from the China Family Panel Studies (CFPS), a nationally representative longitudinal survey funded by the 985 Project and conducted by the Institute of Social Science Survey (ISSS) at Peking University (7). The CFPS sample covers 25 provincial-level administrative regions, including five “large-sample provinces” (Shanghai, Liaoning, Henan, Gansu, and Guangdong) and 20 “small-sample provinces” such as Jiangsu, Zhejiang, Fujian, Jiangxi, Anhui, Shandong, Hubei, and Beijing, collectively representing 94.5% of China's population. The survey uses a multi-stage, stratified sampling design with proportional allocation to ensure national representativeness.

The CFPS collects comprehensive data on individuals, families, and communities, encompassing a wide range of domains such as demographic characteristics, socioeconomic status, and physical and mental health. Surveys are administered through computer-assisted personal interviews, with quality control procedures such as telephone verification, audio audits, and statistical checks implemented to ensure data accuracy (7, 8). The CFPS is conducted biennially and has completed seven waves between 2010 and 2022. Ethical approval was obtained from the Peking University Institutional Review Board, and all participants provided informed consent.

This study utilized data from the 2020 and 2022 waves, focusing on children and adolescents aged 10 to 18 years. Of the initial 6,051 respondents in this age group, those with missing data on PA (*n* = 2,194) or SSB (*n* = 741) were excluded. Additional exclusions were made for missing data on key covariates, including urban/rural residence (*n* = 45), educational level (*n* = 57), sleep duration (*n* = 684), academic pressure (*n* = 1), and interpersonal relationships (*n* = 1). The final analytic sample comprised 2,328 participants (1,047 females), with a mean age of 14.18 ± 2.52 years.

### 2.2 Assessment of physical activity

PA was measured using two items from the 2020 and 2022 CFPS. The first question asked, “Over the past 12 months, how frequently did you engage in physical exercise?” Respondents selected from eight response options: (1) less than once a month on average; (2) more than once a month but less than once a week; (3) 1–2 times per week; (4) 3–4 times per week; (5) 5 or more times per week; (6) once per day; (7) twice or more per day; and (8) never. The second question asked, “How many minutes do you typically exercise each time on average?” and respondents provided a numeric answer.

Based on WHO guidelines (3) and the structure of the survey items, participants were classified as having “sufficient PA” if they engaged in exercise at least five times per week, with each session lasting more than 60 min. Those who did not meet both criteria were classified as having “insufficient PA.”

### 2.3 Assessment of screen-based sedentary behavior

The CFPS assessed five types of SSB: online gaming, online shopping, short video watching, online learning, and WeChat use. For online gaming, shopping, short videos, and online learning, participants were first asked, “In the past week, did you [e.g., play online games]?” If the answer was yes, a follow-up question asked, “Did you do this every day in the past week?”

WeChat use was assessed slightly differently. Participants were first asked, “In the past year, have you used WeChat?” If yes, they were then asked, “How frequently do you post updates about your life to your Moments (similar to Facebook or Instagram)?”

For the purposes of this study, SSB frequency was categorized into three levels:

• “Never” (coded as 1) if the participant answered “no” to the initial question,

• “Occasional” (coded as 2) if the initial response was “yes” but the follow-up was “no,” and

• “Daily” (coded as 3) if both responses were “yes.”

### 2.4 Covariates

Based on previous studies and the availability of data, this study included a range of covariates related to demographic characteristics, lifestyle factors, psychosocial status, and health conditions (9–13). Demographic variables included gender (male = 1, female = 0), age (as a continuous variable ranging from 10 to 18 years), educational level (elementary = 3, junior high = 4, high school = 5), and urban/rural residence (urban = 1, rural = 0). The lifestyle variable considered was sleep duration, with participants categorized as 0 for sleeping ≥8 h per day and 1 for < 8 h. Psychosocial factors included perceived academic pressure (rated from 1 to 5, with higher scores indicating greater pressure) and interpersonal relationships (scored from 0 to 10, with higher scores reflecting better peer relationships). Health status was assessed by self-rated health, coded on a five-point scale where 1 = excellent and 5 = poor. These covariates were selected to adjust for potential confounding factors and are described in detail in Table 1.

**Table 1:** The definitions of the key variables.

**Categories**	**Variables**	**Definition**
Dependent	Physical activity	1 = Sufficient PA (≥5 times/week, ≥60 min each); 2 = Insufficient PA (does not meet both criteria)
Independent	Online gaming	1 = Did not play in the past week; 2 = Played but not daily; 3 = Played daily
	Online shopping	1 = Did not shop in the past week; 2 = Shopped but not daily; 3 = Shopped daily
	Short video watching	1 = Did not watch in the past week; 2 = Watched but not daily; 3 = Watched daily
	Online learning	1 = Did not engage in the past week; 2 = Engaged but not daily; 3 = Engaged daily
	WeChat use	1 = Did not use WeChat in the past year; 2 = Used but did not post Moments; 3 = Used and posted Moments
Controlled	Age	Continuous variable (10–18 years)
	Gender	1 = Male; 0 = Female
	Educational level	3 = Elementary; 4 = Junior high; 5 = High school
	Urban/rural residence	1 = Urban; 0 = Rural
	Sleep time	0 = ≥8 h/day; 1 = < 8 h/day
	Academic pressure	Continuous variable (1–5); higher score = greater pressure
	Interpersonal relationship	Continuous variable (0–10); higher score = better relationships
	Self-assessed health status	1 = Excellent; 2 = Very good; 3 = Good; 4 = Fair; 5 = Poor

### 2.5 Statistical analyses

Descriptive analyses were first conducted to summarize the distribution of SSB and PA. Independent sample *t*-tests were used for continuous variables, and chi-square tests for categorical variables, to examine differences in SSB and PA across various subgroups.

Subsequently, three generalized linear logistic regression models were constructed to examine the associations between different types of SSB and insufficient PA.

•Model 1 included only the control variables: gender, age, educational level, urban/rural residence, academic pressure, interpersonal relationships, and self-rated health status.

•Model 2 added the five SSB variables (online gaming, online shopping, short video watching, online learning, and WeChat use) to Model 1.

•Model 3 further included sleep duration as an additional covariate, building on Model 2.

All statistical analyses were performed using Python (version 3.12, Python Software Foundation, Wilmington, DE, USA), with the aid of libraries including statsmodels, scipy, and matplotlib (14). Statistical significance was determined using a two-tailed *p*-value threshold of 0.05.

## 3 Results

### 3.1 Participant characteristics

Table 2 presents the demographic characteristics of the 2,328 participants included in the final sample. The mean age was 14.18 years (SD = 2.52), and 55.03% were male. Participants were distributed across educational level as follows: 32.56% in elementary school, 35.95% in junior high school, and 31.49% in high school. A total of 48.24% resided in rural areas, and 70.96% reported sleeping at least 8 h per day.

**Table 2:** Characteristics of participants according to cluster of physical activity.

**Variable**	**Category**	**Total no. (%)**	**Sufficient physical activity**		***P*-value from Chi-2/*t*-test**
		**Yes no. (%)**	**No no. (%)**	
Age	Mean (SD)	14.18 (2.52)	14.09 (2.49)	14.23 (2.54)	0.226
AP^a^	Mean (SD)	2.75 (1.03)	2.75 (1.07)	2.75 (1.01)	0.942
IR^a^	Mean (SD)	7.00 (1.90)	7.19 (1.95)	6.91 (1.87)	**0.001**
SH^a^	Mean (SD)	1.95 (0.87)	1.88 (0.89)	1.98 (0.86)	**0.006**
Gender	Female	1,047 (44.97%)	327 (40.67%)	720 (47.24%)	**0.003**
	Male	1,281 (55.03%)	477 (59.33%)	804 (52.76%)	
EL^a^	Elementary	758 (32.56%)	265 (32.96%)	493 (32.35%)	0.444
	Junior high	837 (35.95%)	299 (37.19%)	538 (35.30%)	
	High	733 (31.49%)	240 (29.85%)	493 (32.35%)	
Urban/Rural	Urban	1,205 (51.76%)	397 (49.38%)	808 (53.02%)	0.104
	Rural	1,123 (48.24%)	407 (50.62%)	716 (46.98%)	
Sleep time	≥8 h/day	1,652 (70.96%)	560 (69.65%)	1,092 (71.65%)	0.335
	< 8 h/day	676 (29.04%)	244 (30.35%)	432 (28.35%)	
Independent					
Online gaming	No	914 (39.26%)	327 (40.67%)	587 (38.52%)	0.529
	Occasional	957 (41.11%)	327 (40.67%)	630 (41.34%)	
	Daily	457 (19.63%)	150 (18.66%)	307 (20.14%)	
Online shopping	No	1,485 (63.79%)	513 (63.81%)	972 (63.78%)	0.991
	Occasional	815 (35.01%)	281 (34.95%)	534 (35.04%)	
	Daily	28 (1.20%)	10 (1.24%)	18 (1.18%)	
Short video watching	No	323 (13.87%)	126 (15.67%)	197 (12.93%)	0.114
	Occasional	1,018 (43.73%)	355 (44.15%)	663 (43.50%)	
	Daily	987 (42.40%)	323 (40.17%)	664 (43.57%)	
Online learning	No	1,278 (54.90%)	442 (54.98%)	836 (54.86%)	0.273
	Occasional	684 (29.38%)	224 (27.86%)	460 (30.18%)	
	Daily	366 (15.72%)	138 (17.16%)	228 (14.96%)	
WeChat use	No	330 (14.18%)	105 (13.06%)	225 (14.76%)	0.354
	No Posted	1,965 (84.41%)	685 (85.20%)	1,280 (83.99%)	
	Posted on Moments	33 (1.42%)	14 (1.74%)	19 (1.25%)	
Total			285 (12.24%)	2,043 (87.76%)	

^a^AP, Academic Pressure; IR, Interpersonal Relationship; SH, Self-assessed Health Status; EL, Educational Level.

Bold values indicate *p* < 0.05.

Regarding psychosocial and health-related factors, the mean academic pressure score was 2.75 (SD = 1.03; range: 1–5), the average interpersonal relationship score was 7.00 (SD = 1.90; range: 0–10), and the average self-rated health status was 1.95 (SD = 0.87; lower scores indicate better health).

In terms of SSB, 19.63% of participants reported playing online games daily, while 1.2% shopped online daily. Daily short video watching was reported by 42.4% of participants, 15.72% engaged in online learning daily, and 1.42% reported posting to WeChat Moments.

### 3.2 Univariate analysis

**Figure 1:**
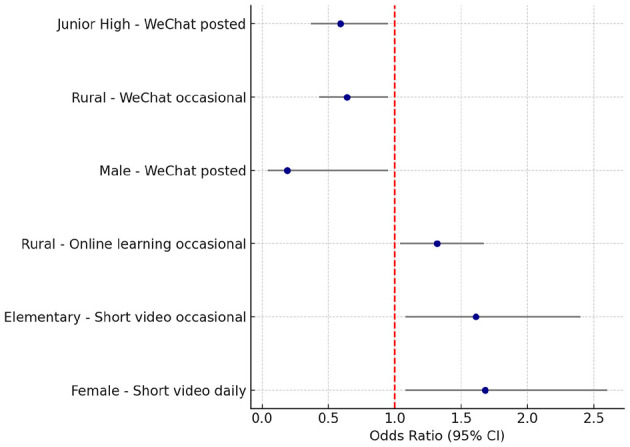
Associations between screen-based sedentary behaviors and insufficient physical activity across subgroups. Forest plot showing ORs and 95% CIs for the associations between SSB and insufficient PA in subgroup analyses. Daily short video watching increased the odds of insufficient PA among females and occasional watching among elementary students. Occasional online learning predicted insufficient PA in rural youth. In contrast, WeChat use showed protective associations among males, junior high students, and rural occasional users.

Table 2 and Figure 1 also compares the characteristics of participants with and without sufficient PA. Significant differences were observed between the two groups in terms of interpersonal relationships, self-rated health status, and gender (*p* < 0.01). Specifically, participants with sufficient PA reported better interpersonal relationships, better perceived health, and were more likely to be male compared to those with insufficient PA. No statistically significant differences were found in age, educational level, urban-rural residence, or sleep duration. Similarly, the frequencies of various types of SSB did not differ significantly between the two groups in the univariate analysis.

### 3.3 Association between screen-based sedentary behaviors and physical activity

Regression models (Table 3) showed that gender, interpersonal relationships, and self-rated health were consistently associated with PA. For example, males were less likely than females to report insufficient PA, better self-rated health predicted higher PA, and stronger social relationships also supported activity. By contrast, none of the five SSB types showed significant associations with PA in the full models. Importantly, their odds ratios clustered around 1.0, indicating little independent explanatory value at the population level. The overall explanatory power of the models remained low (pseudo *R*^2^ = 0.01–0.02), suggesting that broader social and health factors outweigh screen behaviors in predicting PA.

**Table 3:** Associations between screen-based sedentary behaviors and physical activity.

**Variable**	**Category**	**Model 1**		**Model 2**		**Model 3**	
		**OR**	**95% CI**	**OR**	**95% CI**	**OR**	**95% CI**
Age	9	Ref.		Ref.		Ref.	
	Each year increase	1.04	[0.96, 1.13]	1.04	[0.96, 1.14]	1.05	[0.96, 1.14]
AP^a^	1	Ref.		Ref.		Ref.	
	Each level improvement	0.97	[0.89, 1.06]	0.98	[0.90, 1.06]	0.98	[0.90, 1.07]
IR^a^	0	Ref.		Ref.		Ref.	
	Each level improvement	**0.94** ^**^	**[0.89, 0.98]**	**0.94** ^*^	**[0.90, 0.99]**	**0.94** ^*^	**[0.90, 0.99]**
SH^a^	Excellent	Ref.		Ref.		Ref.	
	Each level decline	**1.11** ^*^	**[1.00, 1.23]**	1.11	[1.00, 1.23]	**1.11** ^*^	**[1.00, 1.23]**
Gender	Female	Ref.		Ref.		Ref.	
	Male	**0.77** ^**^	**[0.65, 0.92]**	**0.70** ^***^	**[0.58, 0.85]**	**0.70** ^***^	**[0.58, 0.85]**
EL^a^	Elementary	Ref.		Ref.		Ref.	
	Junior high	0.83	[0.60, 1.15]	0.82	[0.59, 1.14]	0.84	[0.60, 1.16]
	High	0.86	[0.51, 1.45]	0.87	[0.52, 1.47]	0.91	[0.54, 1.54]
Urban/rural	Rural	Ref.		Ref.		Ref.	
	Urban	0.86	[0.72,1.02]	0.87	[0.73, 1.04]	0.87	[0.73, 1.04]
Independent							
Online gaming	No			Ref.		Ref.	
	Occasional			1.14	[0.93, 1.39]	1.13	[0.92, 1.39]
Daily			1.26	[0.97, 1.66]	1.26	[0.96, 1.64]
Online shopping	No			Ref.		Ref.	
	Occasional			0.89	[0.73, 1.09]	0.90	[0.73, 1.00]
Daily			0.83	[0.37, 1.83]	0.83	[0.38, 1.85]
Short video watching	No			Ref.		Ref.	
	Occasional			1.13	[0.87, 1.48]	1.13	[0.86, 1.47]
Daily			1.23	[0.93, 1.63]	1.22	[0.92, 1.61]
Online learning	No			Ref.		Ref.	
	Occasional			1.10	[0.90, 1.34]	1.10	[0.90, 1.34]
Daily			0.86	[0.68, 1.10]	0.86	[0.68, 1.10]
WeChat use	No			Ref.		Ref.	
	No Posted			0.81	[0.62, 1.06]	0.82	[0.62, 1.07]
Posted on Moments			0.56	[0.26, 1.18]	0.56	[0.26, 1.18]
Sleep time	≥8 h/day					Ref.	
	< 8 h/day					0.87	[0.71, 1.06]
Constant		2.18	[0.75, 6.30]	1.98	[0.66, 5.92]	1.94	[0.65, 1.81]
Pseudo *R*^2^		0.01		0.02		0.02	
Observation		2,328		2,328		2,328	

^a^AP, Academic Pressure; IR, Interpersonal Relationship; SH, Self-assessed Health Status; EL, Educational Level.

^*^p < 0.05, ^**^p < 0.01, ^***^p < 0.001.

Bold values indicate *p* < 0.05.

### 3.4 Association between screen-based sedentary behaviors and physical activity in subsamples

Subgroup analyses (Supplementary Tables 1–3; Figure 1) revealed divergent associations across population groups. Short video watching was linked to higher odds of insufficient PA, particularly among females (daily users: OR = 1.68) and elementary students (occasional users: OR = 1.61). In contrast, WeChat use appeared protective, with males, junior-high students, and rural occasional users all showing lower odds of insufficient PA. Online learning demonstrated a risk effect only among rural children and adolescents, where occasional users were more likely to report insufficient PA (OR = 1.32). Additionally, a marginal association between shorter sleep duration (< 8 h/day) and lower odds of insufficient PA was observed among females, suggesting a complex interplay between sleep and activity in this subgroup. While these subgroup effects highlight potential at-risk populations, many estimates had wide confidence intervals and attenuated after false discovery rate correction; thus, the findings should be regarded as exploratory and warrant confirmation in future research with larger samples and pre-specified hypotheses.

## 4 Discussion

This study employed nationally representative data to investigate the associations between various types of SSB and PA among Chinese children and adolescents. The findings revealed that online gaming and online shopping were not significantly associated with PA. In contrast, short video watching was identified as a risk factor for insufficient PA, particularly among females and elementary school students. Online learning was associated with reduced PA levels among rural participants, whereas WeChat use emerged as a protective factor, especially among males, rural participants, and junior high school students.

Online gaming, which primarily relies on visual feedback and user interaction via screens, is often considered a highly immersive form of entertainment. Previous studies have shown that excessive gaming is negatively associated with PA, particularly when it involves traditional sedentary formats that lack physical movement (15). Furthermore, gaming has been identified as a major risk factor for obesity and metabolic syndrome in children and adolescents (16). However, more recent research suggests that active online games—or “exergames”—can promote body movement and effectively reduce sedentary time (17). In the present study, the lack of significant associations may be attributed to the unique characteristics of Chinese children and adolescents, who are frequently subject to strict parental supervision as well as national anti-addiction policies (18). For example, the National Press and Publication Administration of China issued regulations in 2021 restricting minors' access to online games to a maximum of 1.5 h per day, and prohibiting gameplay between 10:00 p.m. and 8:00 a.m. (19). These policy measures, combined with parental monitoring, may have constrained gaming behavior to the extent that it no longer significantly impacts PA. Nevertheless, future research should further distinguish between different types of games to examine their divergent effects on children and adolescents' PA.

Online shopping, a convenient form of digital consumption, typically involves browsing and purchasing items via screens. While excessive online shopping has been associated with reduced PA in adults—particularly when it becomes a primary form of recreation (20)—our findings revealed no significant association among children and adolescents. This may be attributed to the relatively low prevalence of online shopping within this population, who generally lack financial independence and decision-making autonomy, thereby limiting both their engagement in the behavior and its potential impact on PA.

Since the outbreak of COVID-19, online learning has become a dominant form of SSB among children and adolescents. Previous studies have pointed out that online learning often requires students to remain sedentary in front of screens for extended periods, thereby reducing opportunities for PA (21). Moreover, the fast-paced nature of virtual instruction and the lack of structured breaks may further contribute to reductions in overall PA (22). Interestingly, our findings showed that the negative impact of online learning on PA was evident only among rural students. This may be attributed to two key factors. First, in rural online learning environments, break times are less structured, and students often spend recess on additional screen activities instead of PA (23). Second, rural children and adolescents tend to have lower health awareness and weaker exercise habits. In these settings, both families and schools may place less emphasis on the importance of PA, resulting in limited motivation for self-initiated movement (24). In contrast, urban students often have greater access to health knowledge and digital exercise resources—such as fitness apps or online workout classes—which may buffer the sedentary effects of online learning. These findings suggest that future interventions targeting online learning should be tailored specifically to the needs of rural children and adolescents.

Short video platforms have rapidly become a prevalent form of SSB, especially among children and adolescents, due to their brief, entertaining content, algorithm-driven recommendations, and high-speed updates (25). Existing studies have shown that excessive short video watching is associated with reduced PA in adolescents (26). The immersive nature of these platforms often leads users to remain seated for long periods, occupying time that could otherwise be devoted to PA (38). This pattern is consistent with displacement theory, which posits that SSB directly substitute for time that might otherwise be spent in PA (27). In this study, short video watching was identified as a risk factor for insufficient PA, particularly among girls and elementary school students. This may be because these groups tend to have lower intrinsic motivation for PA and weaker self-regulation. By contrast, boys and older students usually possess stronger intentions to engage in PA and greater self-control, enabling them to maintain a more balanced lifestyle. Although adolescents face significant academic pressures, secondary schools typically ensure a basic level of PA through mandatory physical education and extracurricular sports (28). Therefore, the impact of short video use on PA likely depends on a combination of factors including gender, age, self-regulation, and access to PA resources. Future research should examine the psychological and behavioral patterns of short video use among girls and younger children and develop targeted interventions to promote healthier screen habits.

The use of social media platforms represents a unique type of SSB. Although it involves low-intensity physical engagement, its social and interactive nature may indirectly affect PA. Prior research has suggested that online social networking can have both positive and negative effects on PA among children and adolescents. On one hand, platforms that foster peer connection can facilitate outdoor and group activity participation (29). On the other hand, excessive use may reduce real-world social interaction and discourage participation in PA (30). As China's widely used social media platform, WeChat serves not only as a tool for emotional expression but also as a vehicle for organizing group activities. Our findings showed a protective association between WeChat use and insufficient PA among males, rural participants, and junior high school students. This may be partially explained by social facilitation theory, which suggests that interpersonal interactions can foster collective engagement in PA (31). However, this association should be interpreted with caution: WeChat use may serve as a proxy for greater social engagement, better household resources, or higher socioeconomic status, rather than being inherently protective. Reverse causation is also possible, as more active youths might use WeChat to coordinate sporting activities. Moreover, aggregate measures mask purpose-specific differences (e.g., coordination vs. passive browsing), thereby limiting causal inference. Nonetheless, subgroup-specific contexts may help explain the observed associations. One explanation is that in rural areas, despite limited access to PA infrastructure, youth may use WeChat groups and Moments to share information and initiate activity-related plans. Among males, online social interactions through WeChat may translate into offline group-based exercise (32). Similarly, for junior high students—who are in a critical period of social-emotional development—WeChat plays a central role in their peer relationships and may help facilitate collective physical engagement (33).

To the best of our knowledge, this is the first study to investigate the associations between multiple types of SSB and PA among Chinese children and adolescents using nationally representative data. A major strength of this study is its inclusion of subgroup analyses across gender, urban/rural residence, and educations level, which provides nuanced insights into population-specific patterns and risk profiles.

Despite its contributions, this study has several limitations. First, all variables relied on self-reported measures, which may introduce recall and social desirability biases, and the CFPS did not capture detailed time-use data. Future research should incorporate objective measures, such as accelerometers or digital tracking tools, to improve validity. Second, the overall explanatory power of our regression models was relatively low (pseudo *R*^2^ = 0.01–0.02), suggesting that important confounders such as parental education, socioeconomic status, or school-level factors were not fully accounted for. Third, subgroup analyses involved multiple comparisons, but no formal correction was applied, which may increase the risk of chance findings. Finally, as the CFPS is cross-sectional, causal inferences cannot be drawn, and longitudinal research is needed to clarify the mechanisms linking SSB and PA.

## 5 Conclusions

This study identified short video watching as a significant risk factor for insufficient PA among Chinese children and adolescents, particularly among females and elementary school students. Online learning was also linked to reduced PA among rural participants, while WeChat use appeared protective, especially for junior high students, rural participants, and males. These findings underscore the need for subgroup-specific interventions that account for the diverse roles of screen use. Future policies should monitor evolving screen behaviors and implement tailored strategies to mitigate risks and promote PA and health in children and adolescents.

## Data Availability

The datasets presented in this study can be found in online repositories. The names of the repository/repositories and accession number(s) can be found below: The data of the studies is publicly available and could be accessible via website: https://www.isss.pku.edu.cn/cfps/en/index.htm.

## References

[1] Caspersen CJ Powell KE Christenson GM. Physical activity, exercise, and physical fitness: definitions and distinctions for health-related research. Public Health Rep. (1985) 100:126–31. 3920711 PMC1424733

[2] Poitras VJ Gray CE Borghese MM Carson V Chaput J-P Janssen I . Systematic review of the relationships between objectively measured physical activity and health indicators in school-aged children and youth. Appl Physiol. Nutr. Metab. (2016) 41(6 Suppl. 3):S197–239. doi: 10.1139/apnm-2015-0663 27306431

[3] World Health Organization. WHO Guidelines on Physical Activity and Sedentary Behaviour. Geneva: World Health Organization (2020). 33369898

[4] Guthold R Stevens GA Riley LM Bull FC. Global trends in insufficient physical activity among adolescents: a pooled analysis of 298 population-based surveys with 1·6 million participants. Lancet Child Adolesc Health. (2020) 4:23–35. doi: 10.1016/S2352-4642(19)30323-2 31761562 PMC6919336

[5] Ballard M Gray M Reilly J Noggle M. Correlates of video game screen time among males: body mass, physical activity, and other media use. Eat Behav. (2009) 10:161–7. doi: 10.1016/j.eatbeh.2009.05.001 19665099

[6] Melkevik O Torsheim T Iannotti RJ Wold B. Is spending time in screen-based sedentary behaviors associated with less physical activity: a cross national investigation. Int J Behav Nutr Phys Activity. (2010) 7:46. doi: 10.1186/1479-5868-7-46 20492643 PMC3224890

[7] Xie Y Hu J. An introduction to the China Family Panel Studies (CFPS). Chinese Sociol Rev. (2014) 47:3–29. doi: 10.2753/CSA2162-0555470101.2014.11082908

[8] Xie Y Lu P. The sampling design of the China Family Panel Studies (CFPS). Chin J Sociol. (2015) 1:471–84. doi: 10.1177/2057150X15614535 29854418 PMC5973535

[9] Lin H Chang C Liu Z Tan H. The effect of the presence of children on adult smoking behaviour: empirical evidence based on China family panel studies. BMC Public Health. (2020) 20:1448. doi: 10.1186/s12889-020-09543-2 32972391 PMC7513303

[10] Tian F Yang X Xu F Dong R Song Y Fan C . Physical activity and its fluctuations in relation to depressive symptoms: a national longitudinal study among Chinese adults. J Affect Disord. (2024) 347:192–8. doi: 10.1016/j.jad.2023.10.065 37924983

[11] Wang Z Zeng Z. Association between personality characteristics and sleep quality among Chinese middle-aged and older adults: evidence from China family panel studies. BMC Public Health. (2023) 23:2427. doi: 10.1186/s12889-023-17352-6 38053067 PMC10699122

[12] Xue H Fang C Shi J Hu X Qian F. Can preschool out-of-kindergarten tutoring improve approaches to learning for children? Evidence from China Family Panel Studies (CFPS) 2012 to 2020. Sustainability. (2023) 15:1246. doi: 10.3390/su15021246

[13] Zhang X Zhang Y Guo B Chen G Zhang R Jing Q . The impact of physical activity on household out-of-pocket medical expenditure among adults aged 45 and over in urban China: the mediating role of spousal health behaviour. SSM - Popul Health. (2024) 25:101643. doi: 10.1016/j.ssmph.2024.101643 38449524 PMC10915402

[14] Python Software Foundation. Python Language Reference, version 3.11. Available online at: http://www.python.org (Accessed Jan 28, 2023).

[15] Kaya A Türk N Batmaz H Griffiths MD. Online gaming addiction and basic psychological needs among adolescents: the mediating roles of meaning in life and responsibility. Int J Ment Health Addict. (2024) 22:2413–37. doi: 10.1007/s11469-022-00994-9 36643385 PMC9831379

[16] Han T Cho H Sung D Park M-H A. systematic review of the impact of COVID-19 on the game addiction of children and adolescents. Front Psychiatry. (2022) 13:976601. doi: 10.3389/fpsyt.2022.976601 36061296 PMC9435970

[17] Lissak G. Adverse physiological and psychological effects of screen time on children and adolescents: literature review and case study. Environ Res. (2018) 164:149–57. doi: 10.1016/j.envres.2018.01.015 29499467

[18] Wartberg L Kriston L Zieglmeier M Lincoln T Kammerl R A. longitudinal study on psychosocial causes and consequences of Internet gaming disorder in adolescence. Psychol Med. (2019) 49:287–94. doi: 10.1017/S003329171800082X 29622057

[19] National Press and Publication Administration. Notice on Further Strict Management to Effectively Prevent Minors from Online Game Addiction (2021). Available online at: https://www.gov.cn/zhengce/zhengceku/2021-09/01/content_5634661.htm (Accessed Jun 22, 2025).

[20] Tyrväinen O Karjaluoto H. Online grocery shopping before and during the COVID-19 pandemic: a meta-analytical review. Telemat Inform. (2022) 71:101839. doi: 10.1016/j.tele.2022.101839 35607591 PMC9117166

[21] Gallo LA Gallo TF Young SL Moritz KM Akison LK. The impact of isolation measures due to COVID-19 on energy intake and physical activity levels in Australian university students. Nutrients. (2020) 12:1865. doi: 10.3390/nu12061865 32585830 PMC7353248

[22] Mouratidis K Papagiannakis A. COVID-19, internet, and mobility: the rise of telework, telehealth, e-learning, and e-shopping. Sust Cities Soc. (2021) 74:103182. doi: 10.1016/j.scs.2021.103182 34540566 PMC8437688

[23] Forseth B Carlson JA Ortega A Steel C Lancaster B Fitzpatrick LK . O the places rural children will go…to get physical activity: a cross sectional analysis. BMC Public Health. (2025) 25:1188. doi: 10.1186/s12889-025-22442-8 40155954 PMC11954351

[24] Luo Y Li X Zeng Z Fang W. Development prospects and influencing factors of rural sports consumption market. In: Proceedings of the International Conference on Education and Management Science (ICEMS 2014). Lancaster: Destech Publications, Inc. (2014). p. 337–41.

[25] Ye J-H Wu Y-T Wu Y-F Chen M-Y Ye J-N. Effects of short video addiction on the motivation and well-being of Chinese vocational college students. Front Public Health. (2022) 10:847672. doi: 10.3389/fpubh.2022.847672 35619803 PMC9127725

[26] Tian X Bi X Chen H. How short-form video features influence addiction behavior? Empirical research from the opponent process theory perspective. Inform Technol People. (2023) 36:387–408. doi: 10.1108/ITP-04-2020-0186

[27] Ekelund U Luan J Sherar LB Esliger DW Griew P Cooper A. Moderate to vigorous physical activity and sedentary time and cardiometabolic risk factors in children and adolescents. JAMA. (2012) 307:704–12. doi: 10.1001/jama.2012.156 22337681 PMC3793121

[28] Martins J Marques A Sarmento H Carreiro da Costa F. Adolescents' perspectives on the barriers and facilitators of physical activity: a systematic review of qualitative studies. Health Educ Res. (2015) 30:742–55. doi: 10.1093/her/cyv042 26324394

[29] Best P Manktelow R Taylor B. Online communication, social media and adolescent wellbeing: a systematic narrative review. Child Youth Serv Rev. (2014) 41:27–36. doi: 10.1016/j.childyouth.2014.03.001

[30] Woods HC Scott H. #Sleepyteens: social media use in adolescence is associated with poor sleep quality, anxiety, depression and low self-esteem. J Adolesc. (2016) 51:41–9. doi: 10.1016/j.adolescence.2016.05.008 27294324

[31] Bond CF Titus LJ. Social facilitation: a meta-analysis of 241 studies. Psychol Bull. (1983) 94:265–92. doi: 10.1037//0033-2909.94.2.265 6356198

[32] Rodgers RF Slater A Gordon CS McLean SA Jarman HK Paxton SJ . Biopsychosocial model of social media use and body image concerns, disordered eating, and muscle-building behaviors among adolescent girls and boys. J Youth Adolesc. (2020) 49:399–409. doi: 10.1007/s10964-019-01190-0 31907699

[33] Cauberghe V Van Wesenbeeck I De Jans S Hudders L Ponnet K. How adolescents use social media to cope with feelings of loneliness and anxiety during COVID-19 lockdown. Cyberpsychol Behav Soc Netw. (2021) 24:250–7. doi: 10.1089/cyber.2020.0478 33185488

[34] Rodriguez-Ayllon M Cadenas-Sánchez C Estévez-López F Muñoz NE Mora-Gonzalez J Migueles JH . Role of physical activity and sedentary behavior in the mental health of preschoolers, children and adolescents: a systematic review and meta-analysis. Sports Med. (2019) 49:1383–410. doi: 10.1007/s40279-019-01099-5 30993594

[35] van Sluijs EMF Ekelund U Crochemore-Silva I Guthold R Ha A Lubans D . Physical activity behaviours in adolescence: current evidence and opportunities for intervention. Lancet. (2021) 398:429–42. doi: 10.1016/S0140-6736(21)01259-9 34302767 PMC7612669

[36] Aubert S Barnes JD Demchenko I Hawthorne M Abdeta C Abi Nader P . Global matrix 4.0 physical activity report card grades for children and adolescents: results and analyses from 57 countries. J Phys Act Health. (2022) 1:1–29. doi: 10.1123/jpah.2022-0456 36280233

[37] Kim Y Umeda M Lochbaum M Stegemeier S. Physical activity, screen-based sedentary behavior, and sleep duration in adolescents: youth risk behavior survey, 2011–2013. Prev Chronic Dis. (2016) 13:160245. doi: 10.5888/pcd13.160245 27634781 PMC5027845

[38] Zhang D Yang Y Guan M. A cross-lagged analysis of the relationship between short video overuse behavior and depression among college students. Front Psychol. (2024) 15:1345076. doi: 10.3389/fpsyg.2024.1345076 39086426 PMC11289595

