# Supplementary material for: Not all screens are equal: associations between screen-based sedentary behavior and physical activity in Chinese children and adolescents
Source: Front Public Health. 2025 Nov 19;13:1681183. doi: 10.3389/fpubh.2025.1681183 (PMC12672544; doi:10.3389/fpubh.2025.1681183)
Supplement: Supplementary file 1 [file Table_1.docx]

**Supplementary Table 1** Associations between screen-based sedentary behaviors and physical activity: subsamples by gender

|  |  | **Gender** | |
| --- | --- | --- | --- |
|  |  | **Female** | **Male** |
| Age | 9 | Ref. | Ref. |
|  | Each year increase | 1.06 (0.99, 1.13) | 1.02 (0.97, 1.08) |
| AP ^a^ | 1 | Ref. | Ref. |
|  | Each level improvement | 0.95 (0.83, 1.09) | 0.98 (0.88, 1.10) |
| IR ^a^ | 0 | Ref. | Ref. |
|  | Each level improvement | **0.93* (0.86, 1.00)** | 0.95 (0.89, 1.01) |
| SH ^a^ | Excellent | Ref. | Ref. |
|  | Each level decline | 1.15 (0.98, 1.36) | 1.06 (0.93, 1.21) |
| **Independent** |  |  |  |
| Online gaming | No | Ref. | Ref. |
|  | Occasional | 1.20 (0.89, 1.61) | 1.03 (0.77, 1.38) |
|  | Daily | 1.53 (0.90, 2.61) | 1.19 (0.85, 1.68) |
| Online shopping | No | Ref. | Ref. |
|  | Occasional | 0.96 (0.71, 1.29) | 0.84 (0.64, 1.12) |
|  | Daily | 0.59 (0.20, 1.72) | 1.10 (0.32, 3.73) |
| Short video watching | No | Ref. | Ref. |
|  | Occasional | 1.07 (0.71, 1.62) | 1.22 (0.92, 1.63) |
|  | Daily | **1.68* (1.08, 2.60)** | 1.06 (0.78, 1.44) |
| Online learning | No | Ref. | Ref. |
|  | Occasional | 1.12 (0.82, 1.53) | 1.10 (0.84, 1.43) |
|  | Daily | 0.86 (0.59, 1.24) | 0.89 (0.64, 1.24) |
| WeChat use | No | Ref. | Ref. |
|  | No Posted | 0.61 (0.40, 0.94) | 0.97 (0.69, 1.37) |
|  | Posted on Moments | 0.62 (0.93, 1.28) | **0.19* (0.04,0.95)** |
| Sleep time | ≥8 hours/day | Ref. | Ref. |
|  | <8 hours/day | **0.73* (0.54, 1.00)** | 0.97 (0.74, 1.27) |
| Constant |  | 1.9 (0.62, 5.77) | 1.57 (0.62, 3.97) |
| Pseudo R2 |  | 0.037 | 0.014 |
| Observation |  | 1047 | 1281 |

a: AP = Academic Pressure, IR = Interpersonal Relationship, SH = Self-assessed Health Status, EL = Educational Level

b: *p < 0.05, **p < 0.01, ***p < 0.001

**Supplementary Table 2** Associations between screen-based sedentary behaviors and physical activity: subsamples by urban / rural

|  |  | **Urban/rural residence** | |
| --- | --- | --- | --- |
|  |  | **Urban** | **Rural** |
| Age | 9 | Ref. | Ref. |
|  | Each year increase | 1.02 (0.97, 1.09) | 1.03 (0.97, 1.09) |
| AP ^a^ | 1 | Ref. | Ref. |
|  | Each level improvement | 0.91 (0.81, 1.03) | 1.08 (0.95, 1.22) |
| IR ^a^ | 0 | Ref. | Ref. |
|  | Each level improvement | **0.92* (0.86, 0.98)** | 0.96 (0.90, 1.02) |
| SH ^a^ | Excellent | Ref. | Ref. |
|  | Each level decline | 1.12 (0.97, 1.30) | 1.12 (0.97, 1.30) |
| **Independent** |  |  |  |
| Online gaming | No | Ref. | Ref. |
|  | Occasional | 0.96 (0.73, 1.26) | 1.07 (0.81, 1.42) |
|  | Daily | 1.32 (0.93, 1.89) | 0.81 (0.56, 1.16) |
| Online shopping | No | Ref. | Ref. |
|  | Occasional | 1.02 (0.77, 1.37) | 0.87 (0.66, 1.14) |
|  | Daily | 1.23 (0.36, 4.18) | 0.66 (0.23, 1.93) |
| Short video watching | No | Ref. | Ref. |
|  | Occasional | 1.11 (0.78, 1.59) | 1.18 (0.79, 1.75) |
|  | Daily | 1.19 (0.81, 1.74) | 1.35 (0.89, 2.04) |
| Online learning | No | Ref. | Ref. |
|  | Occasional | 1.20 (0.90, 1.59) | **1.32* (1.04, 1.67)** |
|  | Daily | 0.97 (0.68, 1.37) | 1.12 (0.83, 1.49) |
| WeChat use | No | Ref. | Ref. |
|  | No Posted | 1.05 (0.73,1.52) | **0.64* (0.43,0.95)** |
|  | Posted on Moments ^c^ | 0.81 (0.33,1.98) |  |
| Sleep time | ≥8 hours/day | Ref. | Ref. |
|  | <8 hours/day | 0.88 (0.66, 1.18) | 0.83 (0.63, 1.10) |
| Constant |  | 1.89 (0.72,4.99) | 1.72 (0.61,4.86 |
| Pseudo R2 |  | 0.020 | 0.018 |
| Observation |  | 1123 | 1205 |

a: AP = Academic Pressure, IR = Interpersonal Relationship, SH = Self-assessed Health Status, EL = Educational Level

b: *p < 0.05, **p < 0.01, ***p < 0.001

c: Due to the limited number of rural participants who updated their WeChat Moments, these participants were combined with those who used WeChat without updating Moments for analysis.

**Supplementary Table 3** Associations between screen-based sedentary behaviors and physical activity: subsamples by educational level

|  |  | **Educational Level** | | |
| --- | --- | --- | --- | --- |
|  |  | **Elementary** | **Junior High** | **High** |
| Age | 9 | Ref. | Ref. | Ref. |
|  | Each year increase | 0.94 (0.81, 1.09) | 1.11 (0.97, 1.27) | 1.01 (0.85, 1.19) |
| AP ^a^ | 1 | Ref. | Ref. | Ref. |
|  | Each level improvement | 0.96 (0.83, 1.11) | 1.04 (0.90, 1.20) | 0.95 (0.80, 1.13) |
| IR ^a^ | 0 | Ref. | Ref. | Ref. |
|  | Each level improvement | 0.92* (0.86, 0.99) | 0.96 (0.89, 1.04) | 0.92 (0.83, 1.01) |
| SH ^a^ | Excellent | Ref. | Ref. | Ref. |
|  | Each level decline | 1.33** (1.10, 1.62) | 1.14 (0.96, 1.35) | 0.95 (0.78, 1.16) |
| **Independent** |  |  |  |  |
| Online gaming | No | Ref. | Ref. | Ref. |
|  | Occasional | 0.86 (0.62, 1.20) | 1.01 (0.73, 1.41) | 1.24 (0.85, 1.79) |
|  | Daily | 0.7 (0.48, 1.04) | 1.01 (0.67, 1.52) | 1.27 (0.83, 1.95) |
| Online shopping | No | Ref. | Ref. | Ref. |
|  | Occasional | 1.02 (0.65, 1.60) | 1.12 (0.81, 1.54) | 0.80 (0.58, 1.11) |
|  | Daily | 0.24 (0.02, 2.96) | 0.55 (0.15, 2.62) | 1.85 (0.50, 6.84) |
| Short video watching | No | Ref. | Ref. | Ref. |
|  | Occasional | **1.61* (1.08, 2.40)** | 1.36 (0.84, 2.18) | 0.58 (0.33, 1.03) |
|  | Daily | 1.54 (0.97, 2.46) | 1.42 (0.86, 2.32) | 0.78 (0.44, 1.37) |
| Online learning | No | Ref. | Ref. | Ref. |
|  | Occasional | 1.24 (0.87, 1.78) | 0.80 (0.58, 1.10) | 1.41 (0.97, 2.05) |
|  | Daily | 0.91 (0.59, 1.42) | 0.75 (0.49, 1.15) | 0.98 (0.65, 1.49) |
| WeChat use | No | Ref. | Ref. | Ref. |
|  | No Posted | 0.97 (0.68, 1.37) | **0.59* (0.37, 0.95)** | 0.96 (0.32, 2.91) |
|  | Posted on Moments | 1.09 (0.24, 4.88) | 0.82 (0.19, 3.45) | 0.39 (0.09, 1.77) |
| Sleep time | ≥8 hours/day | Ref. | Ref. | Ref. |
|  | <8 hours/day | 1.02 (0.61, 1.72) | 0.78 (0.57, 1.07) | 0.92 (0.67, 1.27) |
| Constant |  | 3.27 (0.52, 20.61) | 0.5 (0.06, 4.14) | 5.69 (0.24, 132.75) |
| Pseudo R2 |  | 0.033 | 0.024 | 0.027 |
| Observation |  | 758 | 837 | 733 |

a: AP = Academic Pressure, IR = Interpersonal Relationship, SH = Self-assessed Health Status, EL = Educational Level

b: *p < 0.05, **p < 0.01, ***p < 0.001
